# FRoG—A New Calculation Engine for Clinical Investigations with Proton and Carbon Ion Beams at CNAO

**DOI:** 10.3390/cancers10110395

**Published:** 2018-10-23

**Authors:** KyungDon Choi, Stewart B Mein, Benedikt Kopp, Giuseppe Magro, Silvia Molinelli, Mario Ciocca, Andrea Mairani

**Affiliations:** 1 Centro Nazionale di Adroterapia Oncologica, 27100 Pavia, Italy; kyungdon.choi@cnao.it (K.C.); giuseppe.magro@cnao.it (G.M.); silvia.molinelli@cnao.it (S.M.); mario.ciocca@cnao.it (M.C.); 2 Department of Physics, Pavia University, 27100 Pavia, Italy; 3 Department of Physics, Heidelberg University, 69120 Heidelberg, Germany; s.mein@dkfz-heidelberg.de; 4 Imaging and Radiation Oncology, German Cancer Research Center (DKFZ), 69210 Heidelberg, Germany; b.kopp@dkfz-heidelberg.de; 5 Radiation Oncology, Heidelberg University Clinic, 69120 Heidelberg, Germany; 6 Heidelberg Ion Beam Therapy Center (HIT), 69120 Heidelberg, Germany

**Keywords:** hadrontherapy, treatment planning, biological dose, MKM, LEM, FRoG, GPU

## Abstract

A fast and accurate dose calculation engine for hadrontherapy is critical for both routine clinical and advanced research applications. FRoG is a graphics processing unit (GPU)-based forward calculation tool developed at CNAO (Centro Nazionale di Adroterapia Oncologica) and at HIT (Heidelberg Ion Beam Therapy Center) for fast and accurate calculation of both physical and biological dose. FRoG calculation engine adopts a triple Gaussian parameterization for the description of the lateral dose distribution. FRoG provides dose, dose-averaged linear energy transfer, and biological dose-maps, -profiles, and -volume-histograms. For the benchmark of the FRoG calculation engine, using the clinical settings available at CNAO, spread-out Bragg peaks (SOBPs) and patient cases for both proton and carbon ion beams have been calculated and compared against FLUKA Monte Carlo (MC) predictions. In addition, FRoG patient-specific quality assurance (QA) has been performed for twenty-five proton and carbon ion fields. As a result, for protons, biological dose values, using a relative biological effectiveness (RBE) of 1.1, agree on average with MC within ~1% for both SOBPs and patient plans. For carbon ions, RBE-weighted dose (D_RBE_) agreement against FLUKA is within ~2.5% for the studied SOBPs and patient plans. Both MKM (Microdosimetric Kinetic Model) and LEM (Local Effect Model) D_RBE_ are implemented and tested in FRoG to support the NIRS (National Institute of Radiological Sciences)-based to LEM-based biological dose conversion. FRoG matched the measured QA dosimetric data within ~2.0% for both particle species. The typical calculation times for patients ranged from roughly 1 to 4 min for proton beams and 3 to 6 min for carbon ions on a NVIDIA^®^ GeForce^®^ GTX 1080 Ti. This works demonstrates FRoG’s potential to bolster clinical activity with proton and carbon ion beams at CNAO.

## 1. Introduction

Radiation therapy requires dose computation on computed tomography (CT) images for each patient with high accuracy and fast calculation time. A clinical treatment planning system (TPS) for proton and carbon ions typically uses an analytical algorithm for dose calculation on a central processing unit (CPU). Recently, graphics processing unit (GPU)-based clinical TPSs have been made available (e.g., Raystation, RaySearch Laboratories, Stockholm, Sweden), promising enhanced calculation speeds for the even more time-consuming Monte Carlo (MC) codes [1]. The benefit of improved precision is clear for the more technologically demanding modalities such as particle therapy. In the near future, compact high-performance systems will become commonplace in the clinic, making routine integration of advanced physical and biophysical models feasible [1,2,3].

Presently, the commercial TPS used during clinical operation at CNAO (Centro Nazionale Adroterapia Oncologica [4]), Syngo-VC13 (Siemens AG, Mannheim, Germany), is a CPU-based system. Syngo-VC13 calculates biological dose by applying a fixed relative biological effectiveness (RBE) of 1.1 [5] for proton beams and the biological model LEM I (Local Effect Model [6]) for carbon ion beams. Syngo, as well as the other analytical systems, in comparison to MC approaches, has limitations in handling patient heterogeneities and small fields with and without the range shifter (RS) especially with proton beams [7]. Additionally, calculation features for clinically relevant biophysical quantities are often missing from such programs, such as dose-averaged linear energy transfer (LET_d_) distributions in water for proton beams or RBE-weighted dose (D_RBE_) for carbon ions with other biological models including the modified MKM (Microdosimetric Kinetic Model [8]). The modified MKM is applied at the carbon ion therapy facility NIRS (National Institute of Radiological Sciences) in Japan for carbon ion therapy planning [9]. At CNAO, the fractionation scheme used at NIRS has been employed requiring the introduction of a conversion between NIRS-based to LEM-based D_RBE_, as calculated by Syngo [10,11].

In order to overcome these limitations, we have introduced a new calculation platform, FRoG (Fast Recalculation on GPU), to perform fast physical and biological calculations for proton and carbon ion beam therapy. The FRoG project is a collaborative effort between the CNAO facility in Pavia, Italy, and the Heidelberg Ion Beam Therapy Center (HIT) [12] in Heidelberg, Germany. FRoG was designed to perform fast and accurate calculations, applying an analytical pencil beam algorithm on GPU to calculate physical/biological dose and LET_d_ distributions. For proton beams, clinicians and medical physicists can assess both dose and LET_d_ distributions (and eventually D_RBE_ applying variable RBE scheme [13,14]) to further scrutinize plans for acceptance or potential re-planning purposes within minutes. For carbon ions, the user can quickly access MKM-based D_RBE_ values and compare them with the converted LEM-based D_RBE_ predictions by the use of a scaling factor introduced in Fossati et al. [10]. In presence of large hot or cold spots in the tumor, the plan can be readily re-optimized taking into account the FRoG MKM-based D_RBE_ values.

In this work, the benchmarks performed are presented for both proton and carbon ions in preparation for FRoG’s integration into clinical activity. SOBPs and patient cases have been calculated and compared against FLUKA MC predictions [15,16,17], which serves as the reference MC code at CNAO and was used to generate the physical database of Syngo [18,19,20,21]. Various LET_d_ maps and LEM vs. MKM D_RBE_ distributions for patients treated with proton and carbon ion beams, respectively, are presented. Prior to patient treatment, the plan from Syngo is verified dosimetrically by performing field-specific quality assurance (QA) [18]. In this work, we have used twenty-five field-specific QA for each beam modality and we have compared them against FRoG predictions.

## 2. Results

### 2.1. Comparison of FLUKA- and FRoG-Calculated Spread Out Bragg Peaks

Syngo-optimized SOBPs without beam modifiers (i.e., range shifter), have been calculated with FRoG and compared against FLUKA predictions. For protons, the results are summarized in Table 1 in terms of percent D_RBE_ difference of the RBE-weighted dose-volume histogram (D_RBE_VH) parameters D_RBE,50_, D_RBE,2_, and D_RBE,98_ for an SOBP with equal sides of 3 cm (target volume 27 cm^3^) and 6 cm (target volume of 216 cm^3^) at different depths: 3 cm, 12 cm, and 21 cm. For a definition of these parameters see Section 4.2.1. The RBE was assumed to be 1.1.

FRoG matches well with the MC predictions. Considering all resultant values (D_RBE,50_, D_RBE,2_, and D_RBE,98_), in terms of absolute variation, FRoG reproduces FLUKA results within 0.7% on average. The observed maximum variation was 1.1% for D_RBE,98_ for an SOBP of side 3 cm at 21 cm depth and for an SOBP of side 6 cm at 12 cm depth.

Carbon ions D_RBE_VH statistics for the target volume for the six studied SOBPs are summarized in Table 2 and Table 3 for LEM and MKM, respectively. For 27 cm^3^ (216 cm^3^) target volumes, applying the LEM model, percent D_RBE_ differences for D_RBE,50_, D_RBE,2_, and D_RBE,98_ are 0.4% (0.7%), −1.9% (−0.6%), and −0.1% (2.0%), respectively. These results are obtained by averaging over the three SOBP depths.

For 27 cm^3^ (216 cm^3^) target volumes, applying the MKM model, percent D_RBE_ differences for D_RBE,50_, D_RBE,2_, and D_RBE,98_ are −1.1% (−0.4%), −2.2% (−1.5%), and −1.7% (0.3%), respectively. Overall, the FRoG biological dose calculations with the studied SOBPs match FLUKA predictions within 2.5%. However, variations larger than or close to 3% have been found for LEM D_RBE,98_ at 12 cm (216 cm^3^ target volume) and MKM D_RBE,98_ at 21 cm (27 cm^3^ target volume).

### 2.2. Comparison of FLUKA- and FRoG-Calculated Patient Cases

#### 2.2.1. Proton Beams

**Figure 1:**
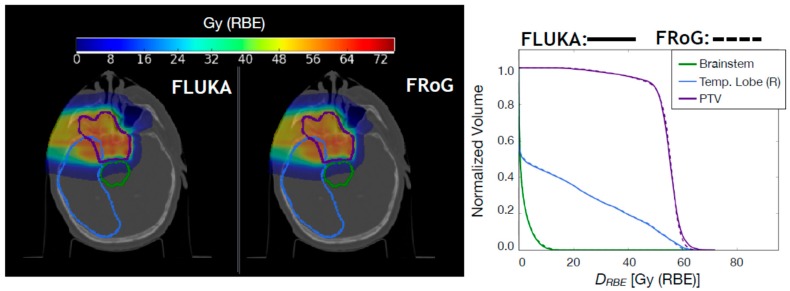
FLUKA and FRoG recalculated D_RBE_ distributions for a proton head case without range shifter (RS) are displayed in the left and middle panel, respectively, together with the contours of three regions of interest: the planning target volume (PTV), brainstem, and right temporal lobe. The D_RBE_VH for the regions of interest (ROIs) are shown in the right panel.

**Figure 2:**
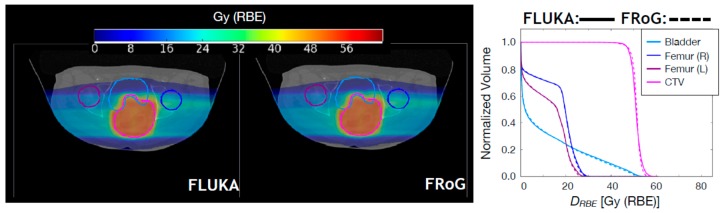
FLUKA and FRoG recalculated D_RBE_ distributions for a proton pelvic case are displayed in the left and middle panel, respectively, together with the contours of four regions of interest: the clinical target volume (CTV), right/left femur, and bladder. The D_RBE_VH for the ROIs are shown in the right panel.

For proton beams, fifteen CNAO patient cases have been recalculated with FRoG and FLUKA: five head and neck (H&N) without RS, five H&N with RS, and five pelvic cases without RS. The FRoG and FLUKA D_RBE_ predictions (RBE = 1.1) have been compared in terms of variation of D_RBE,50_, D_RBE,2_, and D_RBE,98_ in the target volume and in terms of γ-analysis [22,23]. In Figure 1 and Figure 2, D_RBE_ distributions and D_RBE_VH comparisons between FLUKA and FRoG are presented for a H&N case without RS and a pelvic case, respectively.

A summary of variations in target D_RBE,50_, D_RBE,2_, and D_RBE,98_, with respect to MC calculations for proton patient cases is reported in Table 4. Average percent D_RBE_VH differences between FLUKA simulation and FRoG over the whole patient set were within ~0.7%. γ-pass rates, i.e., percent of points for which the γ-index is ≤1, were 98.4%, 96.0%, and 97.2% for the H&N without RS, H&N with RS, and pelvic patient cases, respectively. The overall γ-pass rate was 97.2%.

The average calculation time for the ten H&N cases was approximately 90 s ± 22 s, with a maximum of 3 min and 57 s (PTV of 300 cm^3^, three beams with RS) and minimum of 44 s (PTV of 15.5 cm^3^, three beams with RS). For the pelvic cases, the average calculation time was approximately 175 s ± 60 s ranging from a maximum of 6 min and 35 s (PTV of 2134 cm^3^, two beams) to a minimum of 1 min and 2 s (PTV of 91 cm^3^, two beams). The reported runtime also includes calculating LET_d_ (as well as RBE) distributions which are typically unavailable in a commercial TPS.

**Figure 3:**
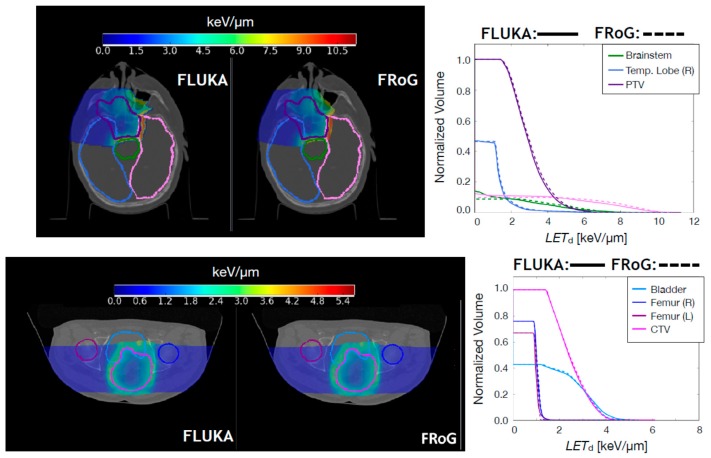
Upper panels: FLUKA and FRoG recalculated LET_d_ distributions for a proton head case are displayed in the left and middle panel, respectively, together with the contours of four regions of interest: PTV, brainstem, and right and left temporal lobe. The LET_d_VH for the ROIs are shown in the right panel. Lower panels: FLUKA and FRoG recalculated LET_d_ distributions for a proton pelvic case are displayed in the left and middle panel, respectively, together with the contours of four regions of interest: CTV, right and left femur, and bladder. The LET_d_VH for the ROIs are shown in the right panel.

In Figure 3, LET_d_ maps and LET_d_-volume-histograms (LET_d_VH) calculated by FRoG and FLUKA for the two patients of Figure 1 and Figure 2 are shown. For the head case, the left temporal lobe was examined in order to quantify the high LET_d_ component found, as expected, in the distal region of the field. For the PTV, FRoG and FLUKA LET_d_ values match within 0.05 keV/µm. The FRoG calculated mean LET_d_ is approximately 3.0 keV/µm while the LET_d,2_ is 5.7 keV/µm, where LET_d,2_ is the LET_d_ received by 2% of the volume. For the other ROIs, LET_d,2_ are 6.4 keV/µm, 3.0 keV/µm, and 9.5 keV/µm, for the brainstem and the right and left temporal lobe, respectively. These values agree with the FLUKA predictions within ~0.3 keV/ µm. For the pelvic case, the mean LET_d_ for the CTV is 2.5 keV/µm while the LET_d,2_ is 4.0 keV/µm. For the bladder, LET_d,2_ is 4.1 keV/µm while for the lower value of LET_d,2_ for the femurs is ~1.2 keV/µm. These values agree with the FLUKA predictions within ~0.2 keV/µm for organs at risk (OAR) and ~0.05 keV/µm for the CTV.

#### 2.2.2. Carbon Ion Beams

**Figure 4:**
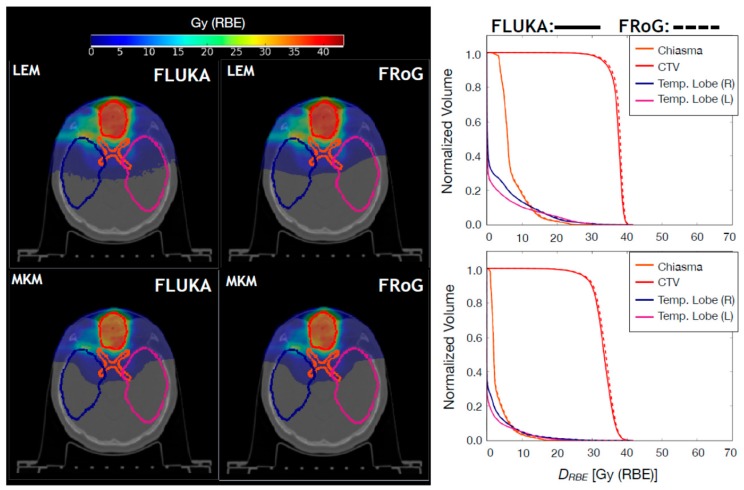
Upper panels: FLUKA and FRoG recalculated LEM-based D_RBE_ distributions for a ^12^C ion head case are displayed in the left and middle panel, respectively, together with the contours of four representative regions of interest: CTV, brainstem, and left/right temporal lobe. The LEM-based D_RBE_VH for the ROIs are shown in the right panel. Lower panels: FLUKA and FRoG recalculated MKM-based D_RBE_ distributions for a ^12^C ion head case are displayed in the left and middle panel, respectively, together with the contours of four regions of interest: CTV, brainstem, and left/right temporal lobe. The MKM-based D_RBE_VH for the ROIs are shown in the right panel.

**Figure 5:**
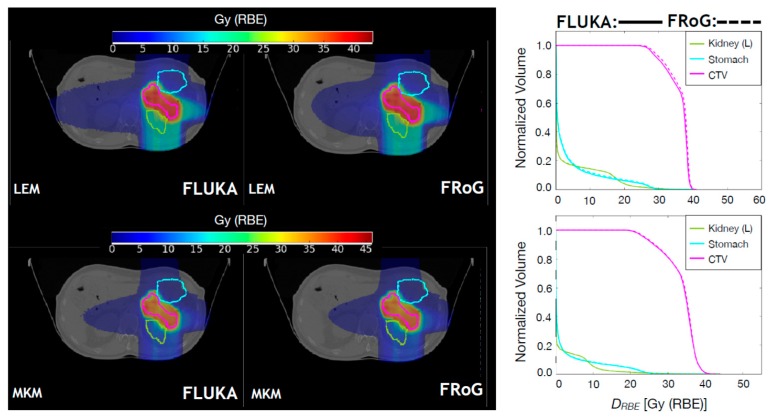
Upper panels: FLUKA and FRoG recalculated LEM-based D_RBE_ distributions for a ^12^C ion pancreatic patient case are displayed in the left and middle panel, respectively, together with the contours of three representative regions of interest: CTV, left kidney and stomach. The LEM-based D_RBE_VH for the ROIs are shown in the right panel. Lower panels: FLUKA and FRoG recalculated MKM-based D_RBE_ distributions for a ^12^C ion pancreatic patient case are displayed in the left and middle panel, respectively, together with the contours of three representative regions of interest: CTV, left kidney, and stomach. The MKM-based D_RBE_VH for the ROIs are shown in the right panel.

For carbon ion beams, ten Syngo LEM-optimized CNAO patient cases were recalculated using FRoG and FLUKA: five H&N and five pelvic cases. The H&N cases include fields with and without RS. Resultant FLUKA and FRoG LEM/MKM-based D_RBE_ distributions are compared. In Figure 4 and in Figure 5, FLUKA and FRoG LEM/MKM D_RBE_ distributions and D_RBE_VH comparisons for a H&N case and a pelvic case, respectively, are shown. In Table 5, percent D_RBE_VH differences between FLUKA MC simulations and FRoG for the target in terms of D_RBE,2_, D_RBE,50_, and D_RBE,98_ are reported for both MKM and LEM D_RBE_ values. Average percent differences over the whole patient set were within 2.5% for both LEM and MKM-based D_RBE_, respectively. γ-pass rates were ~99% for both LEM and MKM in both H&N and pelvic cases.

The average calculation time including absorbed dose, LEM and MKM D_RBE_ for the five H&N cases was about 388 s ± 91 s, ranging from 4 min and 11 s (PTV of 25 cm^3^, 2 beams) to 11 min and 30 s (PTV of 204 cm^3^, two beams). For the pelvic cases, the average calculation time was about 205 s ± 60 s, ranging from 1 min and 33 s (PTV of 40 cm^3^, one beam) to 5 min and 50 s (PTV of 172 cm^3^, one beam).

### 2.3. Comparison against Dosimetric Data

For patient QA, FRoG dose calculations showed a high level of agreement, with the mean and standard deviation of the difference between calculated and measured dose over the twenty-five fields of 1.96% ± 0.79% and 2.21% ± 0.92%, for p and ^12^C, respectively. The Syngo-calculated values were 2.71% ± 1.25% and 2.57% ± 0.84%, for p and ^12^C, respectively. The improved agreement compared to Syngo is due to the employment of a triple Gaussian parameterization in FRoG instead of a double Gaussian model as in Syngo [24] and an improved handling of the RS for proton beams. In FRoG, facility-specific triple Gaussian parameterizations were implemented taking into account RS thickness and RS distance from the patients (or phantoms) entry point. Conversely, as explained previously [7], Syngo numerically accounts for the corresponding water equivalent thickness for radiological depth calculation, but the beam widening due to the RS is only covered by a single Gaussian scattering model [25]. For carbon ions, Syngo neglects the additional scattering of the beams in the RS while FRoG considers a beam energy-dependent parameterization of the beam widening due to the RS. The typical calculation time for a patient QA field is roughly 1 to 2 min depending on particle type, depth, and target volume.

## 3. Discussion

In this paper, benchmarks of FRoG against MC predictions for protons and carbon ions have been performed in both homogeneous (SOBP in water) and heterogeneous scenarios (patient cases). In general, on average discrepancies in terms of D_RBE_ values have been found to be within 1% and 2.5% for proton and carbon ions, respectively. For carbon ions, variations larger than or close to 3% have been found for LEM and for MKM. The steep dose gradients of carbon ions make D_RBE,98_ a less robust metric in the evaluation of the target coverage. Additionally, we have analyzed the D_RBE,95_ (biological dose received by 95% of the PTV) and found an improved agreement with a variation of 1.94% for LEM D_RBE,95_ at 12 cm (side 6 cm) and −1.20% MKM D_RBE,95_ at 21 cm (side 3 cm). In general, the worsening of the agreement in the case of carbon ions is due to the limitations of handling the lateral dose distributions with a simple triple Gaussian approach [24] as well as the assumption that, for biological calculations, lateral variations of the mixed radiation field are not explicitly considered [26]. In order to overcome the latter, an approach similar to the one suggested by Inaniwa and Kanematsu [26] could be implemented. Nevertheless, the FRoG agreement with the MC has been considered highly satisfactorily keeping in mind the larger discrepancies, up to 5–10%, found using commercial TPS [7,27].

In terms of physical dose distributions for carbon ions, FRoG and FLUKA match on average within 1.4% for SOBPs and within 1.6% for patient cases (see Supplementary Material Table S1), in line with results presented previously [24] where calculations of SOBPs in homogenous settings using FRoG were within 1.3% of FLUKA calculations, while analysis of patient case calculations exhibited net deviations in the target within about 1.5%.

For the proton H&N patient cases, presence of the RS marginally reduces the agreement, with the γ-pass rate decreasing from 98.4% to 96.0% with use of the RS. Nonetheless, the high level of agreement found confirms that the RS handling in FRoG is sound, as described in Section 2.3.

For proton beams, LET_d_ values have been found matching the MC predictions within 0.3 keV/µm in both target and organs at risk. This agreement is important when using FRoG for a retrospective study of clinical outcomes in proton therapy. A FRoG user could efficiently perform the analysis carried out in a previous paper [28], determining, for example, if areas of normal tissue damage indicated by post-treatment image changes are associated with increased LET_d_ values.

**Figure 6:**
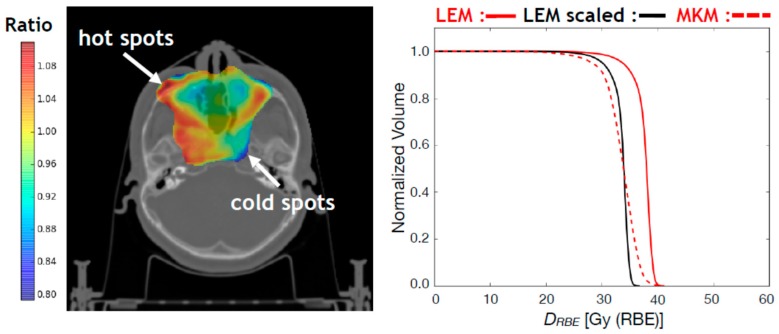
Left panel: ratio between FRoG-calculated MKM D_RBE_ and LEM D_RBE_ multiplied by the clinical scaling value (see the next for more details) for the ^12^C ion patient shown in Figure 4. Only the values for the CTV are displayed. Right panel: FRoG-calculated LEM and MKM D_RBE_VH for the CTV are displayed with a red solid line and red dashed line, respectively. The D_RBE_VH for the LEM D_RBE_ values rescaled by the clinical scaling factor is displayed with a black solid line.

Regarding the carbon ion biological dose, a conversion scheme is implemented at CNAO allowing to plan a NIRS-based median biological dose to the target starting from a prescribed LEM-based biological dose [10,11]. This, however, does not assure a satisfactory coverage in certain cases resulting in hot or cold spots in the biological dose distributions due to the different particle type, LET and tissue dependencies of the two biological models. As an example, in the left panel of Figure 6, the ratio between FRoG-calculated MKM D_RBE_ and LEM D_RBE_, multiplied by the clinical scaling value for the patient shown in Figure 4, is displayed only in the region of the CTV. For this patient, the FRoG-calculated scaling factor (D_RBE,LEM,50_/D_RBE,MKM,50_ for the CTV) is 1.12 which matches the expected one of about 1.10 for 4.3 Gy (RBE) LEM per fraction scheme [10]. If a constant scaling factor were enough to modulate LEM-based biological dose to MKM-based biological dose, the ratio values reported in the left panel of Figure 6 should be close to 1. Instead, hot and cold spots have been found in the CTV as summarized in the D_RBE_VH shown in the right panel of Figure 6. In this context, the accessibility within few minutes of a FRoG MKM-based biological dose could help medical physicists in adjusting the LEM-based constraints, resulting in a more homogenous MKM biological dose mitigating hot and cold spots in the target.

For the studied patient cases, FRoG’s total calculation time was approximately 2 min for proton beams and 5 min for carbon ion beams. Further improvements could be made to FRoG’s calculation speed by upgrading the hardware and optimizing the code structure. For our calculations, we have used a consumer grade GPU card (NVIDIA Corporation, Santa Clara, CA, USA NVIDIA^®^ GeForce^®^ GTX 1080 Ti) as available at CNAO. For understanding the impact of upgrading the GPU card on the calculation time reduction, we have run the most time-consuming case presented in this work, i.e., a H&N carbon ion case (total time: 11 min and 30 s, PTV of 204 cm^3^, two beams) with a high-end graphics card (NVIDIA Corporation, Santa Clara, CA, USA NVIDIA^®^ Tesla^®^ V100) available at HIT. The time reduction was approximately 3.5-fold with a new total calculation time of about 3 min and 20 s.

In terms of dosimetric comparisons, considering both protons and carbon ions, FRoG agreed with measurements better than Syngo, with a 0.56% improvement, on average, in reproducing patient-specific QA measurements. The additional differences of about 2% found with respect to the experimental data could be due to uncertainties involved in the dose delivery process and in the water phantom positioning as described previously [18].

Here, the FRoG platform has been introduced at CNAO and it has been benchmarked successfully against MC prediction and dosimetric measurements for proton and carbon ion beams. Due to its accuracy and fast calculation time in comparison to the MC code, FRoG could be used for retrospective study of large patient cohorts as well as in clinical treatment planning workflow taking into account LET_d_ distributions in proton plans and MKM-based D_RBE_ in carbon ion plans. Organs at risk constraints resulting from toxicity studies of the long-term follow up of Japanese treatments were adopted at CNAO without any correction for the RBE model. This approach is overly conservative and it is currently under evaluation for critical structures such as the optic nerve, brainstem, and rectum.

## 4. Materials and Methods

### 4.1. FRoG Workflow and Design Information

In regard to the input physics database, FRoG handles depth dose distributions and lateral dose parametrizations generated with FLUKA simulated data [18,19,20,29]. As described previously [24], physical and biophysical quantities were simulated and scored depth-wise in water. For the physical dose, lateral evolution was additionally scored radially. Subsequently, lateral dose evolution parameterization as a function of depth using a triple Gaussian model was performed using the MINUIT package [30] in ROOT [31], as described previously [32].

**Figure 7:**
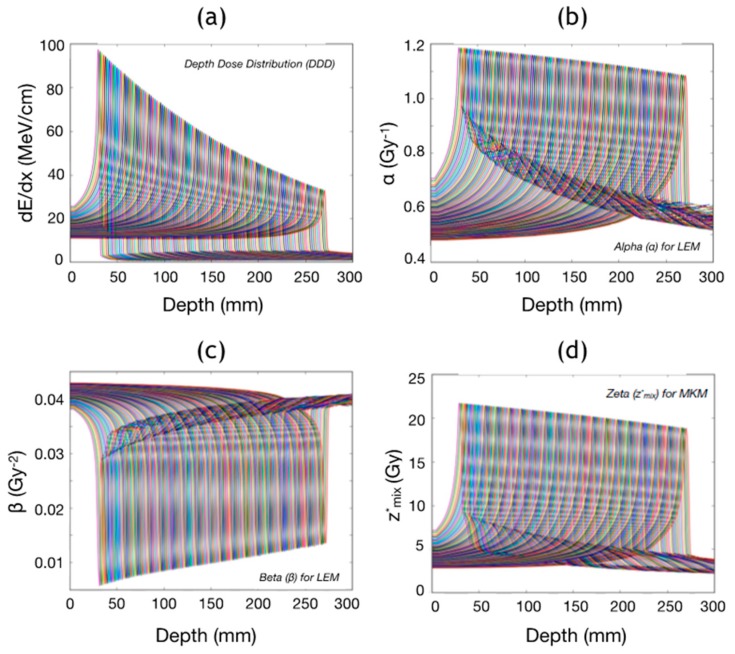
CNAO carbon ion depth-dependent database for all beam energies: (**a**) depth-dose distribution, (**b**) alpha for LEM, (**c**) beta for LEM, and (**d**) z*_mix_ for MKM.

For biological calculations, MC-derived depth-dependent databases for LET_d_, z*_mix_, alpha and beta are available. For carbon ions, z*_mix_ is used for MKM-based calculations [8,33] while alpha and beta for LEM ones [6,34]. Alpha and beta are the dose-averaged linear (alpha) and quadratic (beta) parameters necessary for the LEM while z*_mix_ is the dose-averaged saturation-corrected dose-mean specific energy of the domain delivered in a single event needed by the MKM. FRoG’s depth-dependent database for physical and biological calculations with carbon ion beams at CNAO are shown in Figure 7. The physical and biological input parameters employed for generating the database are explained in detail in previous works [33,34].

FRoG employs a pencil beam algorithm with GPU optimized raytracing [35]. Raytracing is performed with the resolution of the planning CT, whereas dose can be calculated on a downsized grid, following the clinical procedure at CNAO. For head treatments with proton beams, dose calculation is performed on a 2 × 2 × 2 mm^3^ grid with a 0.98 × 0.98 × 2 mm^3^ CT resolution, while for pelvic treatments, dose calculation is performed on a 3 × 3 × 3 mm^3^ grid with a 0.98 × 0.98 × 2 mm^3^ CT resolution. For carbon ions, for the studied cases, dose calculation is performed on a 2 × 2 × 2 mm^3^ grid with a 0.98 × 0.98 × 2 mm^3^ CT resolution. Pencil beam splitting was incorporated into FRoG’s framework following the mathematical procedures of beamlet superposition [36] handling variable lateral dose evolution in the presence of anatomical heterogeneity. In a two-dimensional grid space, the splitting method involves a superposition of N equally-spaced sub-Gaussians (bounded by ±3.5 σ) with equivalent FWHM but variable weighting. For protons ~350 subsplits were used while for carbon ions, ~350 and ~100 subsplits, were applied for patient cases in the H&N and in the pelvic region, respectively. In case of calculations in water phantoms, no splitting is needed. For each beam spot in the patient plan, source-to-surface distance was calculated and full width at half maximum (FWHM) in air projected to the entrance were interpolated using experimentally measured FWHM values for each ion, beam energy, foci, and treatment room as in clinical practice at CNAO using Syngo [20].

The FRoG dose calculation engine adopts a triple Gaussian distribution model for handling lateral dose distribution as a function of the water equivalent depth [24]. For carbon ion therapy, there are two biological models applied clinically, the modified MKM and LEM I. These models are implemented in FRoG and the two D_RBE_ distributions are calculated at the same time of the physical dose. The implementation of the LEM and MKM biological calculations have been performed following the approaches described in the literature [6,8,9,27,33,34]. For proton beams, dose-averaged LET in water is calculated as described previously [27] but weighting only primary protons and secondary protons, deuterons, and tritons. This allows to quickly access D_RBE_ values applying LET-based RBE models [13].

### 4.2. Benchmark of the FRoG Predictions with Proton and Carbon Ion Beams

#### 4.2.1. Comparison against FLUKA Predictions

Six SOBPs for both proton and carbon ions have been optimized with Syngo using RBE = 1.1 for protons and LEM-based optimization for carbon ions [6,7]. The target volumes had cubic shapes with sides of 3 cm or 6 cm. They were contoured in water with the proximal face positioned at a depth of 6 cm, 12 cm, and 21 cm. The optimization goal was homogeneous target coverage to 2 Gy (RBE) for both proton and carbon ions. The results are analyzed in terms of percent D_RBE_ difference of D_RBE,50_, D_RBE,2_, and D_RBE,98_. D_RBE,50_, D_RBE,2_, and D_RBE,98_ represent the biological dose received by 50%, 2%, and 98% of the planning target volume (PTV) in the cumulative D_RBE_VH, respectively. To evaluate the capability of FRoG in handling heterogeneities in clinically relevant scenarios, twenty-five patients previously treated at CNAO have been recalculated. Fifteen patients treated with proton beams (10 H&N, five pelvic cases) and ten with carbon ions (five H&N, five pelvic cases). We have included the most clinically representative targets and beam configurations. Five proton H&N patients have been selected with 3 cm RW3 range shifter, always positioned at 10 cm distance from the patient’s skin. In this way, we could quantify the FRoG predictions when an RS for proton beams was employed. γ-values have been calculated for the regions in the D_RBE_ distributions with a 10% low-dose threshold of the maximum MC D_RBE_. The chosen criteria were 2 mm as distance-to-agreement (DTA) and 2% as D_RBE_ difference.

The FLUKA MC code [15,16,17] was selected to perform forward plan recalculations to benchmark FRoG predictions. FLUKA represents the gold standard for clinical calculations in particle therapy at CNAO [18,19,20].

Patients records were obtained with informed consent and handled following the Helsinki Declaration. All methods were approved and followed applicable guidelines and regulations of CNAO. Considering the retrospective nature of the study, clearing from the ethical review committee was not required. All records were anonymized prior to the study.

#### 4.2.2. Comparison against Dosimetric Data

For experimentally validating FRoG results, dosimetric verification of patient plans in water for proton and carbon ion beams has been performed following the same procedure as for patient quality assurance at CNAO [18]. Twenty-five fields of patient-specific QA for each beam modality have been acquired and compared against FRoG predictions. The measurements were performed using a water phantom (MP3-P T41029, PTW, Freiburg, Germany). The dosimetric system consists of a three-dimensional stack of pin point ionization chambers (ICS, PinPoint Ionization Chambers, TM31015, PTW, Freiburg, Germany), mounted on a robotic arm and positioned inside the water phantom [37]. The position of the block with the ICs can be remotely controlled with a step size of 0.1 mm. The dose distribution for each beam has been measured using 12 ICs simultaneously with the ICs connected to a 12-channel electrometer (MULTIDOS^®^, PTW, Freiburg, Germany). According to the CNAO QA protocol, results are accepted if the mean and the standard deviation of [(measured dose − calculated dose)/maximum field dose] over a data set of 12 points are both within ±5% and below 5%, respectively [18].

## 5. Conclusions

Through a multi-institutional collaboration, we introduce a unique software, FRoG, for rapid and robust dose calculation on GPU for protons and carbon ions at CNAO. As opposed to the commercial TPS used in the clinic, FRoG is a sandbox environment for radiation therapy research, providing the means to develop and evaluate sophisticated models for effective dose (e.g.,: MKM and LEM), as well as physical quantities such as LET_d_. Benchmark tests for the FRoG dose calculation engine using both SOBPs and patient cases were performed against FLUKA MC and dosimetric data, demonstrating satisfactorily agreement. Future efforts with FRoG include retrospective analysis of a large patient cohort to investigate clinical outcome, as well refinement of both physical and biological models in hadrontherapy.

## Figures and Tables

**Table 1:** Percent RBE-weighted dose (D_RBE_) difference comparison for RBE-weighted dose-volume histogram (D_RBE_VH) between FRoG and Monte Carlo (MC) for proton beams.

Percent Difference of	Target Volume	3 cm	12 cm	21 cm
D_RBE,50_	27 cm^3^ 216 cm^3^	0.67% 0.59%	0.59% 0.61%	0.88% 0.71%
D_RBE,2_	27 cm^3^ 216 cm^3^	0.91% −0.60%	−0.09% 0.08%	0.47% 0.73%
D_RBE,98_	27 cm^3^ 216 cm^3^	0.52% 0.46%	0.43% 1.10%	1.07% 0.47%

**Table 2:** Percent D_RBE_ difference comparison for Local Effect Model (LEM) D_RBE_VH between FRoG and MC for ^12^C ions.

Percent Difference of	Target Volume	3 cm	12 cm	21 cm
D_RBE,50_	27 cm^3^ 216 cm^3^	0.93% 0.58%	0.39% 0.64%	−0.01% 1.02%
D_RBE,2_	27 cm^3^ 216 cm^3^	−1.01% −1.14%	−2.45% −0.65%	−2.27% 0.02%
D_RBE,98_	27 cm^3^ 216 cm^3^	−2.19% 2.14%	−0.18% 2.95%	2.03% 0.78%

**Table 3:** Percent D_RBE_ difference comparison for Microdosimetric Kinetic Model (MKM) D_RBE_VH between FRoG and MC for ^12^C ions.

Percent Difference of	Target Volume	3 cm	12 cm	21 cm
D_RBE,50_	27 cm^3^ 216 cm^3^	−1.13% −0.46%	−2.10% −0.41%	−2.97% −0.26%
D_RBE,2_	27 cm^3^ 216 cm^3^	−0.19% −0.68%	−0.90% −1.41%	−2.11% −2.48%
D_RBE,98_	27 cm^3^ 216 cm^3^	0.81% −0.30%	−2.38% 1.91%	−3.86% −0.84%

**Table 4:** Average percent variation of target D_RBE,50_, D_RBE,2_, and D_RBE,98_, with respect to MC calculations for proton patient cases. The values represent the mean ± standard error of the mean.

Percent Difference of	Total	H&N No RS	H&N with RS	Pelvic
D_RBE,50_	0.68% ± 0.21%	1.05% ± 0.32%	0.34% ± 0.50%	0.65% ± 0.31%
D_RBE,2_	−0.57% ± 0.41%	−0.91% ± 0.55%	−0.84% ± 1.05%	0.02% ± 0.67%
D_RBE,98_	0.41% ± 0.56%	1.25% ± 0.42%	−0.79% ± 1.55%	0.77% ± 0.81%

**Table 5:** Average percent variation of target D_RBE,50_, D_RBE,2_, and D_RBE,98_, with respect to MC calculations for carbon ion patient cases applying LEM and MKM biological models. The values represent the mean ± standard error of the mean.

Percent Difference of	Biological Model	Total	H&N	Pelvic
D_RBE,50_	LEM MKM	0.96% ± 0.33% 1.46% ± 0.30%	0.52% ± 0.52% 1.23% ± 0.30%	1.40% ± 0.38% 1.69% ± 0.50%
D_RBE,2_	LEM MKM	0.97% ± 0.30% 1.81% ± 0.46%	0.93% ± 0.62% 2.57% ± 0.78%	1.01% ± 0.32% 1.05% ± 0.36%
D_RBE,98_	LEM MKM	2.23% ± 0.34% 2.36% ± 0.30%	2.80% ± 0.65% 2.93% ± 0.46%	1.67% ± 0.18% 1.79% ± 0.23%

## Supplementary Materials

The following are available online at http://www.mdpi.com/2072-6694/10/11/395/s1, Table S1: Average percent variation in D_50_, D_2_ and D_98_ of the target for FRoG dose calculation with respect to MC for carbon ion patient cases. The values represent the mean ± standard error of the mean.

## References

[1] Saini J., Maes D., Egan A., Bowen S.R., James S.S., Janson M., Wong T., Bloch C. (2017). Dosimetric evaluation of a commercial proton spot scanning Monte-Carlo dose algorithm: Comparisons against measurements and simulations. Phys. Med. Biol..

[2] Jia X., Schümann J., Paganetti H., Jiang S.B. (2012). GPU-based fast Monte Carlo dose calculation for proton therapy. Phys. Med. Biol..

[3] Qin N., Pinto M., Tian Z., Dedes G., Pompos A., Jiang S.B., Parodi K., Jia X. (2017). Initial development of goCMC: A GPU-oriented fast cross-platform Monte Carlo engine for carbon ion therapy. Phys. Med. Biol..

[4] Rossi S. (2015). The national centre for oncological hadrontherapy (CNAO): Status and perspectives. Phys. Med..

[5] International Commission on Radiation Units & Measurements Prescribing, Recording and Reporting Proton-Beam Therapy (*ICRU Report* 78). https://icru.org/home/reports/prescribing-recording-and-reporting-proton-beam-therapy-icru-report-78.

[6] Scholz M., Kellerer A.M., Kraft-Weyrather W., Kraft G. (1997). Computation of cell survival in heavy ion beams for therapy. Radiat. Environ. Biophys..

[7] Magro G., Molinelli S., Mairani A., Mirandola A., Panizza D., Russo S., Ferrari A., Valvo F., Fossati P., Ciocca M. (2015). Dosimetric accuracy of a treatment planning system for actively scanned proton beams and small target volumes: Monte Carlo and experimental validation. Phys. Med. Biol..

[8] Inaniwa T., Furukawa T., Kase Y., Matsufuji N., Toshito T., Matsumoto Y., Furusawa Y., Noda K. (2010). Treatment planning for a scanned carbon beam with a modified microdosimetric kinetic model. Phys. Med. Biol..

[9] Inaniwa T., Kanematsu N., Matsufuji N., Kanai T., Shirai T., Noda K., Tsuji H., Kamada T., Tsujii H. (2015). Reformulation of a clinical-dose system for carbon-ion radiotherapy treatment planning at the National Institute of Radiological Sciences, Japan. Phys. Med. Biol..

[10] Fossati P., Molinelli S., Matsufuji N., Ciocca M., Mirandola A., Mairani A., Mizoe J., Hasegawa A., Imai R., Kamada R. (2012). Dose prescription in carbon ion radiotherapy: A planning study to compare NIRS and LEM approaches with a clinically-oriented strategy. Phys. Med. Biol..

[11] Molinelli S., Magro G., Mairani A., Matsufuji N., Kanematsu N., Inaniwa T., Mirandola A., Russo S., Mastella E., Hasegawa A. (2016). Dose prescription in carbon ion radiotherapy: How to compare two different RBE-weighted dose calculation systems. Radiother. Oncol..

[12] Haberer T., Debus J., Jäkel O., Schulz-Ertner D., Weber U. (2004). The Heidelberg ion therapy center. Radiother. Oncol..

[13] Giovannini G., Böhlen T.T., Cabal G., Bauer J., Tessonnier T., Frey K., Debus J., Mairani K., Parodi A. (2016). Variable RBE in proton therapy: Comparison of different model predictions and their influence on clinical-like scenario. Radiat. Oncol..

[14] Mairani A., Dokic I., Magro G., Tessonnier T., Bauer J., Böhlen T.T., Ciocca M., Ferrari A., Sala P.R., Jäkel O. (2017). A phenomenological relative biological effectiveness approach for proton therapy based on an improved description of the mixed radiation field. Phys. Med. Biol..

[15] Ferrari A., Sala P.R., Fassò A., Ranft J. FLUKA: A Multi-Particle Transport Code. https://www.slac.stanford.edu/pubs/slacreports/reports16/slac-r-773.pdf.

[16] Böhlen T.T., Cerutti F., Chin M.P.W., Fassò A., Ferrari A., Ortega P.G., Mairani A., Sala P.R., Smirnov G., Vlachoudis V. (2014). The FLUKA Code: Developments and Challenges for High Energy and Medical Applications. Nucl. Data Sheets.

[17] Battistoni G., Bauer J., Böhlen T.T., Cerutti F., Chin M.P.W., Dos Santos Augusto R., Ferrari A., Ortega G., Kozlowska W., Magro G. (2016). The FLUKA Code: An Accurate Simulation Tool for Particle Therapy. Front. Oncol..

[18] Molinelli S., Mairani A., Mirandola A., Vilches Freixas G., Tessonnier T., Giordanengo S., Parodi K., Ciocca M., Orecchia R. (2013). Dosimetric accuracy assessment of a treatment plan verification system for scanned proton beam radiotherapy: One-year experimental results and Monte Carlo analysis of the involved uncertainties. Phys. Med. Biol..

[19] Mairani A., Böhlen T.T., Schiavi A., Tessonnier T., Molinelli S., Brons S., Battistoni G., Parodi K., Patera V. (2013). A Monte Carlo-based treatment planning tool for proton therapy. Phys. Med. Biol..

[20] Mirandola A., Molinelli S., Vilches Freixas G., Mairani A., Gallio E., Panizza D., Russo S., Ciocca M., Donetti M., Magro G. (2015). Dosimetric commissioning and quality assurance of scanned ion beams at the Italian National Center for Oncological Hadrontherapy. Med. Phys..

[21] Parodi K., Mairani A., Sommerer F. (2013). Monte Carlo-based parametrization of the lateral dose spread for clinical treatment planning of scanned proton and carbon ion beams. J. Radiat. Res..

[22] Low D.A., Harms W.B., Mutic S., Purdy J.A. (1998). A technique for quantitative evaluation of dose distributions. Med. Phys..

[23] Wendling M., Zijp L.J., McDermott L.N., Smit E.J., Sonke J.J., Mijnheer B.J., van Herk M. (2007). A fast algorithm for gamma evaluation in 3D. Med. Phys..

[24] Mein S., Choi K., Kopp B., Tessonnier T., Bauer J., Ferrari A., Haberer T., Debus J., Abdollahi A., Mairani A. (2018). Fast robust dose calculation on GPU for high-precision ^1^H, ^4^He, ^12^C and ^16^O ion therapy: The FRoG platform. Sci. Rep..

[25] Szymanowski H., Oelfke U. (2002). Two-dimensional pencil beam scaling: An improved proton dose algorithm for heterogeneous media. Phys. Med. Biol..

[26] Inaniwa T., Kanematsu N. (2014). A trichrome beam model for biological dose calculation in scanned carbon-ion radiotherapy treatment planning. Phys. Med. Biol..

[27] Bauer J., Sommerer F., Mairani A., Unholtz D., Farook R., Handrack J., Frey K., Marcelos T., Tessonnier T., Ecker S. (2014). Integration and evaluation of automated Monte Carlo simulations in the clinical practice of scanned proton and carbon ion beam therapy. Phys. Med. Biol..

[28] Peeler C.R., Mirkovic D., Titt U., Blanchard P., Gunther J.R., Mahajan A., Mohan R., Grosshans D.R. (2016). Clinical evidence of variable proton biological effectiveness in pediatric patients treated for ependymoma. Radiother. Oncol..

[29] Parodi K., Mairani A., Brons S., Hasch B.G., Sommerer F., Naumann J., Jäkel O., Haberer T., Debus J. (2012). Monte Carlo simulations to support start-up and treatment planning of scanned proton and carbon ion therapy at a synchrotron-based facility. Phys. Med. Biol..

[30] James F., Roos M. (1975). Minuit—A system for function minimization and analysis of the parameter errors and correlations. Comput. Phys. Commun..

[31] Brun R., Rademakers F. (1997). ROOT—An object oriented data analysis framework. Nucl. Instrum. Methods Phys. Res. A.

[32] Tessonnier T., Böhlen T.T., Ceruti F., Ferrari A., Sala P., Brons S., Haberer T., Debus J., Parodi K., Mairani A. (2017). Dosimetric verification in water of a Monte Carlo treatment planning tool for proton, helium, carbon and oxygen ion beams at the Heidelberg Ion Beam Therapy Center. Phys. Med. Biol..

[33] Magro G., Dahle T.J., Molinelli S., Ciocca M., Fossati P., Ferrari A., Inaniwa T., Matsufuji N., Ytre-Hauge K.S., Mairani A. (2017). The FLUKA Monte Carlo code coupled with the NIRS approach for clinical dose calculations in carbon ion therapy. Phys. Med. Biol..

[34] Mairani A., Brons S., Cerutti F., Fassò A., Ferrari A., Krämer M., Parodi K., Scholz M., Sommerer F. (2010). The FLUKA Monte Carlo code coupled with the local effect model for biological calculations in carbon ion therapy. Phys. Med. Biol..

[35] De Greef M., Crezee J., Van Eijk J.C., Pool R., Bel A. (2009). Accelerated ray tracing for radiotherapy dose calculations on a GPU. Med. Phys..

[36] Russo G., Attili A., Battistoni G., Bertrand D., Bourhaleb F., Cappucci F., Ciocca M., Mairani A., Milian F.M., Molinelli S. (2016). A novel algorithm for the calculation of physical and biological irradiation quantities in scanned ion beam therapy: The beamlet superposition approach. Phys. Med. Biol..

[37] Karger C.P., Jäkel O., Hartmann G.H., Heeg P. (1999). A system for three-dimensional dosimetric verification of treatment plans in intensity-modulated radiotherapy with heavy ions. Med. Phys..

