# Supplementary material for: FRoG—A New Calculation Engine for Clinical Investigations with Proton and Carbon Ion Beams at CNAO
Source: Cancers (Basel). 2018 Oct 23;10(11):395. doi: 10.3390/cancers10110395 (PMC6266031; doi:10.3390/cancers10110395)
Supplement: Supplementary file 1 [file cancers-10-00395-s001.pdf]

Supplementary Materials: FRoG – A New Calculation Engine for Clinical Investigations with Proton and Carbon Ion Beams at CNAO

KyungDon Choi, Stewart B Mein, Benedikt Kopp, Giuseppe Magro, Silvia Molinelli, Mario Ciocca and Andrea Mairani

Table S1. Average percent variation in D_{50}, D_2 and D_{98} of the target for FRoG dose calculation with respect to MC for carbon ion patient cases. The values represent the mean ± standard error of the mean.

Percent Difference of	Total	H&N	Pelvic
D_{50}	1.58% ± 0.18%	1.49% ± 0.32%	1.67% ± 0.21%
D_2	0.84% ± 0.32%	0.73% ± 0.59%	0.95% ± 0.36%
D_{98}	2.22% ± 0.29%	2.61% ± 0.46%	1.82% ± 0.26%

A tabulated summary of D_{50}, D_2, and D_{98} variation in the target, for FRoG dose calculation with respect to MC for carbon ion patient cases, is reported in supplementary table S1. D_{50}, D_2, and D_{98} represent the physical dose received by 50%, 2%, and 98% of the target volume in the cumulative dose-volume histogram (DVH), respectively. Average percent DVH differences between FLUKA simulation and FRoG over the whole patient set were within ~1.6%, ~0.8% and ~2.2%, for D_{50}, D_2, and D_{98}, respectively. The worsening of the agreement for D_{98} between FRoG and FLUKA is due to the steep dose gradients in the physical dose distributions, making D_{98} a less robust metric in the evaluation of the target coverage. Averaging over the results for D_{50}, D_2, and D_{98}, FLUKA and FRoG physical dose predictions match within 1.6%.
